# Advancements in bone marrow biopsy: the role of omics and artificial intelligence in hematologic diagnostics

**DOI:** 10.3389/fmed.2026.1772478

**Published:** 2026-03-24

**Authors:** Maryam Alwahaibi, Nasar Alwahaibi

**Affiliations:** College of Medicine and Health Sciences, Sultan Qaboos University, Muscat, Oman

**Keywords:** artificial intelligence, bone marrow biopsy, digital pathology, hematologic malignancies, omics technologies, precision hematology

## Abstract

Bone marrow biopsy remains central to the diagnosis, classification, and monitoring of hematologic disorders, providing essential morphologic and immunophenotypic information. However, conventional assessment is limited by interobserver variability and an inability to fully capture the molecular and spatial complexity of marrow pathology. Recent advances in multi-omics technologies, including genomic, epigenomic, transcriptomic, proteomic, lipidomic, metabolomic, and microbiomic, together with artificial intelligence (AI), are reshaping bone marrow evaluation. These approaches generate high-dimensional datasets that reveal disease-specific molecular signatures, refine diagnostic classification, improve risk stratification, and inform therapeutic decision-making. In parallel, AI-driven image analysis enhances objectivity, reproducibility, and integration of morphologic and molecular data across both biopsy histology and aspirate cytology. Despite their transformative potential, clinical translation remains challenged by issues of standardization, cost, data integration, and regulatory validation. This mini-review summarizes emerging applications of omics technologies and AI in bone marrow biopsy diagnostics, highlights current limitations, and discusses future directions toward integrated, data-driven precision hematology.

## Introduction

Bone marrow biopsy is a cornerstone of diagnosing and managing a wide spectrum of hematologic disorders, including leukemias, lymphomas, myelodysplastic syndromes, myeloproliferative neoplasms, plasma cell dyscrasias, and marrow failure conditions (1). Conventional diagnostic modalities, including histomorphology, immunohistochemistry, flow cytometry, and cytogenetics, remain essential for classifying disease entities, assessing severity, and guiding initial treatment decisions (2). Despite their diagnostic value, these methods offer only a partial snapshot of the complex biological processes within the marrow. Overlapping morphological features, interobserver variability, and limited ability to capture molecular heterogeneity can lead to diagnostic uncertainty, difficulty predicting progression, and challenges in tailoring therapy (3).

Traditional histopathology in bone marrow biopsy diagnosis is limited by several inherent challenges. Interpretation of histologic features is largely subjective, leading to well-recognized inter- and intra-observer variability among pathologists (4–6). The diagnostic process is time-consuming and labor-intensive, particularly in complex cases or when multiple biopsies are required, which can delay definitive diagnosis and clinical decision-making (7). Certain bone marrow and bone-related diseases exhibit indirect, heterogeneous, or ambiguous morphologic features, especially in rare or dedifferentiated tumors, making accurate diagnosis difficult without highly specialized expertise (8). These challenges are compounded by a global shortage of expert bone pathologists, resulting in increased workload, risk of diagnostic inconsistency, and limited access to specialist interpretation in resource-constrained settings (9, 10).

Advances in omics technologies have begun to overcome these limitations by enabling high-resolution interrogation of the marrow at genetic, transcriptomic, proteomic, metabolomic, and spatial levels (11–14). Genomic sequencing reveals clonal mutations and evolutionary trajectories; transcriptomic profiling uncovers dysregulated pathways; proteomics and metabolomics characterize functional alterations; and single-cell and spatial omics reconstruct the cellular ecosystems and microenvironmental interactions that drive hematologic disease. These approaches provide multilayered insights that complement, refine, and sometimes redefine traditional diagnostic categories (15).

In parallel, artificial intelligence (AI) has emerged as a powerful tool in hematopathology (16–19). Deep learning models can identify subtle morphological abnormalities, quantify features objectively, and improve reproducibility, while machine-learning algorithms integrate imaging, molecular data, and clinical variables to support risk stratification and prognostication (20–23). Multimodal AI systems are now bridging the gap between morphology and molecular biology, creating unified diagnostic frameworks capable of capturing disease complexity in ways previously unattainable.

**Figure 1:**
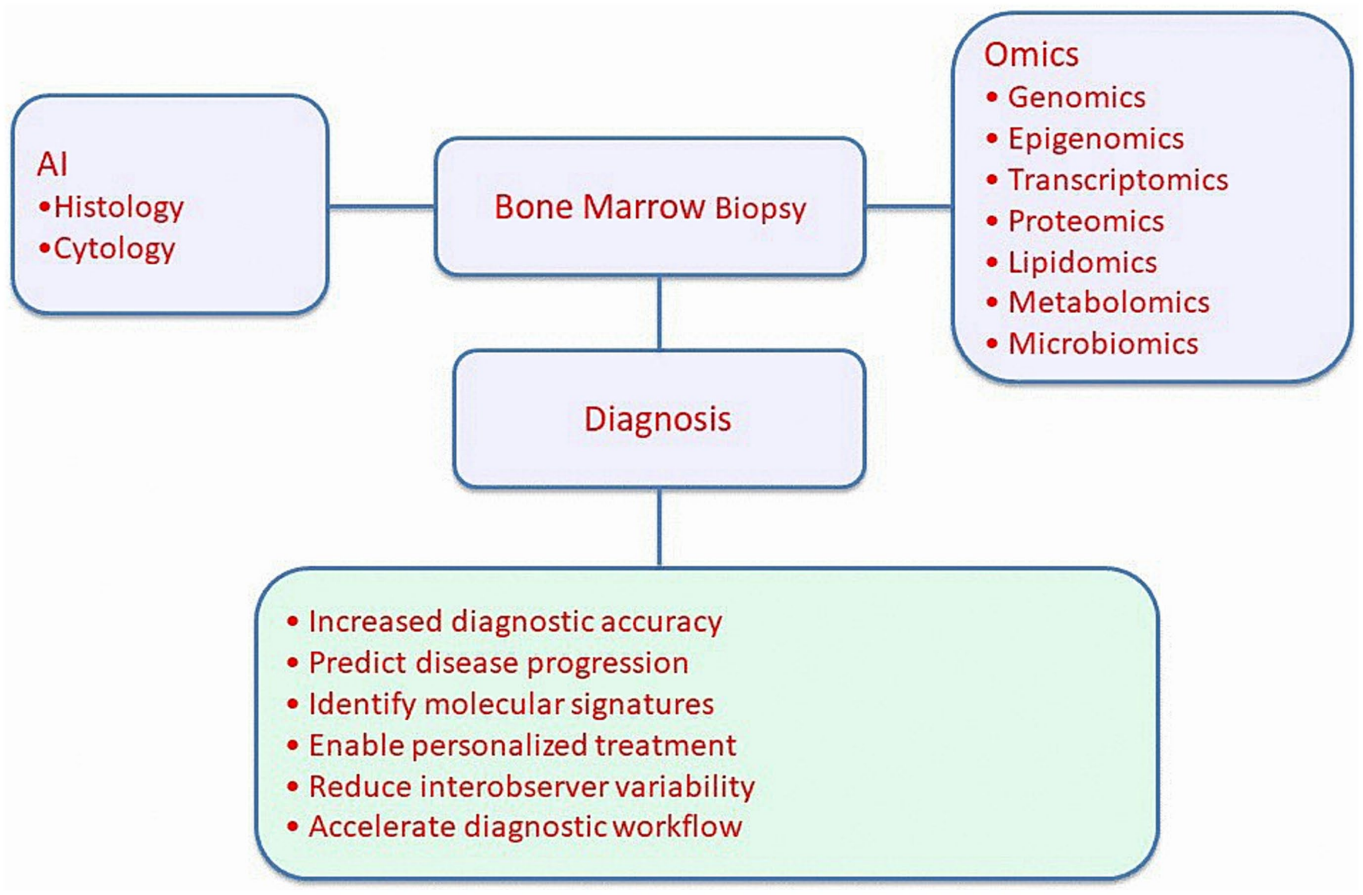
Integration of omics technologies and artificial intelligence in bone marrow biopsy diagnostics.

Together, omics technologies and AI are transforming bone marrow biopsy from a largely morphology-based test into a comprehensive platform for precision hematology (24). By revealing molecular drivers, mapping cellular heterogeneity, and predicting clinical behavior, these innovations promise to enhance diagnostic accuracy, personalize treatment strategies, and deepen our understanding of marrow pathophysiology (25). As integration into clinical practice accelerates, bone marrow examination is poised to become not only a diagnostic tool but also a window into dynamic disease biology, supporting more informed and individualized patient care. An overview of how artificial intelligence and multi-omics technologies integrate with bone marrow biopsy histology and cytology to enhance diagnostic accuracy, prognostication, and workflow efficiency is illustrated in Figure 1.

## Omics technologies in bone marrow diagnosis

Multi-omics approaches have transformed bone marrow diagnostics by enabling comprehensive, multidimensional analysis of the molecular and cellular landscape underlying hematologic disorders, extending far beyond the limits of conventional morphology-based assessment (26). By interrogating genomic, epigenomic, transcriptomic, proteomic, lipidomic, metabolomic, and microbiome-derived features, omics technologies provide deeper insight into disease mechanisms, clonal evolution, and microenvironmental remodeling. Integration of these molecular layers allows refinement of diagnostic classification, detection of minimal residual disease, and identification of therapeutic vulnerabilities, supporting more precise risk stratification and personalized treatment strategies across a wide spectrum of bone marrow diseases (27).

Genomic technologies encompass a wide range of tools used to analyze the structure and function of DNA, providing deep insight into the genetic architecture of hematologic diseases (28). Genomics has transformed bone marrow diagnostics by enabling detection of recurrent mutations associated with clonal hematopoiesis, leukemogenesis, and therapeutic response. Approaches such as targeted sequencing, whole-exome sequencing, and genome-wide association studies (GWAS) help refine disease classification and identify high-risk variants, as demonstrated by large cohort studies linking specific single nucleotide polymorphisms (SNPs) with altered disease susceptibility (29, 30).

Transcriptomics provides a global view of RNA expression profiles within bone marrow cells (31). Modern RNA-sequencing captures gene expression changes, splice variants, and fusion transcripts with high sensitivity (32). Transcriptomic studies have helped identify pathways associated with disease progression, clonal evolution, and inflammatory signaling by profiling expression patterns across diverse hematopoietic and stromal populations (33, 34).

Epigenomics, which investigates reversible modifications to DNA and histones across the genome (35), offers an additional layer of biological insight. Epigenetic mechanisms such as DNA methylation and histone acetylation regulate gene expression programs critical for hematopoietic differentiation and malignant transformation (36). Altered methylation patterns have been associated with dysregulated lineage commitment and aberrant marrow microenvironmental signaling in hematologic disorders (37, 38).

Lipidomics provides complementary insight into bone biology by identifying lipid species that regulate membrane structure, intracellular signaling, and stromal–hematopoietic interactions (39–41). When integrated with broader multi-omics approaches, lipidomic profiling has revealed distinct molecular signatures across diverse bone-related diseases, highlighting disruptions in the skeletal and marrow microenvironment that affect mesenchymal progenitors, osteoblasts, osteoclasts, chondrocytes, immune cells, and specialized stromal subsets (42–46). These disease-specific lipid and molecular patterns illuminate how altered cellular communication, microenvironmental remodeling, and inflammatory signaling drive pathological bone states and influence disease progression.

Proteomics expands this understanding by mapping protein abundance, interactions, and post-translational modifications within the marrow niche (47–49). Proteomic analyses have revealed alterations in extracellular matrix composition, cytokine networks, and signaling pathways that shape malignant hematopoiesis and niche remodeling (50, 51). Key regulatory proteins involved in angiogenesis, cellular proliferation, and immune modulation contribute to disease progression and therapeutic response (52).

Microbiomics research has shown that systemic immune function and inflammation, both influenced by gut microbiota, can indirectly impact marrow homeostasis and hematologic disease progression (53, 54). Dysbiosis-related immune dysregulation may contribute to marrow failure, cytokine imbalance, and treatment response variability (55).

Metabolomics further characterizes bone marrow biology by quantifying metabolites that reflect upstream genomic and proteomic dysregulation (56, 57). Metabolite profiling has identified markers of inflammation, oxidative stress, and aberrant metabolic states that contribute to hematologic disease pathophysiology and may serve as therapeutic targets (58).

A summary of the integrated multi-omics domains and their key contributions to bone marrow diagnostics, including genomics, epigenomics, transcriptomics, proteomics, metabolomics, microbiomics, and lipidomics, is presented in Table 1. The table highlights the representative studies within each domain and outlines their principal insights and relevance to advancing molecular understanding and diagnostic precision in bone marrow and skeletal disorders.

**Table 1:** Integrated multi-omics approaches in bone marrow diagnostics.

Omics domain/technology	Key contributions/insights	Relevance to bone marrow diagnostics	Ref
Genomics	Genome-wide and targeted sequencing approaches; identification of SNPs, disease-associated variants, and regulatory elements influencing cell fate	Enables detection of clonal hematopoiesis, leukemogenic mutations, and inherited susceptibility variants; refines disease classification and risk stratification	(28–30)
Transcriptomics	Bulk and single-cell RNA-seq, transcriptome quantification, fusion detection, splice variant identification	Defines transcriptional programs of hematopoietic and stromal subsets; maps inflammatory and differentiation pathways underlying progression, clonal evolution, and niche remodeling	(31–34)
Epigenomics	Analysis of DNA methylation, histone modifications, chromatin remodeling pathways (Polycomb, COMPASS); epigenetic control of lineage specification	Reveals disrupted differentiation programs and microenvironmental signaling in marrow disorders; identifies mechanisms driving malignant transformation and stem cell dysfunction	(35–38)
Lipidomics	Mass spectrometry–based lipid profiling; integration of lipidomics with multi-omics and spatial analyses	Characterizes lipid species influencing membrane biology, signaling, and stromal–hematopoietic communication; reveals lipid-driven remodeling of bone and marrow microenvironments in disease	(39–46)
Proteomics	Protein abundance and interaction profiling, extracellular matrix characterization, cytokine and signaling pathway analysis	Identifies dysregulated ECM components, angiogenic factors, immune mediators, and signaling proteins contributing to marrow remodeling, malignant hematopoiesis, and therapeutic response	(47–52)
Microbiomics	Microbiome–immune interaction analysis; study of microbial metabolites influencing systemic inflammation	Highlights gut–marrow axis mechanisms; shows how dysbiosis alters systemic immunity, cytokine signaling, marrow homeostasis, and treatment response variability	(53–55)
Metabolomics	Mass spectrometry–based metabolite profiling; metabolic pathway mapping	Detects metabolic signatures associated with oxidative stress, inflammation, hypoxia, and altered energy states—key processes in marrow dysfunction and hematologic disease biology	(56–58)

## AI in bone marrow biopsy diagnosis

AI has become a powerful adjunct in hematopathology, transforming both bone marrow biopsy (histology) and bone marrow aspirate (cytology) evaluation. By providing objective, quantitative, and reproducible analysis, AI reduces the subjectivity and variability inherent in traditional microscopy (59). Across a wide spectrum of diagnostic tasks, including fibrosis grading, megakaryocyte assessment, mutation-associated morphology, plasma cell quantification, lineage recognition, and dysplasia detection, AI systems enhance diagnostic precision, streamline workflows, and deepen insight into the biological underpinnings of hematologic diseases. Together, AI-driven approaches in bone marrow histology and cytology support more accurate disease classification, prognostication, and monitoring, while facilitating scalable and standardized diagnostic practice in both routine and resource-limited settings (60).

## AI in bone marrow histology

AI enables precise and quantitative evaluation of bone marrow fibrosis, improving the detection and monitoring of reticulin changes and supporting accurate myeloproliferative neoplasm (MPN) subtyping. Machine learning–based fibrosis indices also help identify patients at risk of disease progression, refining prognostication in MPNs (61).

AI-driven analysis of megakaryocyte morphology provides automated, reproducible feature extraction that supports reliable MPN diagnosis and differentiation between key subtypes. These abstracted morphologic representations aid clinical interpretation and facilitate monitoring of disease evolution and treatment response (62). Convolutional neural network models can detect subtle morphologic signatures linked to genetic mutations, cytogenetic abnormalities, and prognostic categories. By integrating high-dimensional histologic features with molecular correlates, such as those associated with TET2 mutations or del(5q) MDS, AI enhances diagnostic precision and supports morphology–genotype interpretation in myelodysplastic syndromes (MDS) and MPN evaluation (63).

Deep learning approaches also improve quantification of plasma cells, offering precise and consistent measurements that closely align with expert assessment. These models reduce variability inherent in manual estimation and can be deployed on whole-slide images through workflow-friendly digital platforms, strengthening the diagnosis of plasma cell neoplasms (64). Beyond specific disease classifications, AI models streamline cell detection and classification tasks. A synchronized deep autoencoder enables simultaneous identification and categorization of cells, improving accuracy, reducing computational burden, and enhancing efficiency in bone marrow slide analysis (65).

AI-based image segmentation further standardizes bone marrow cellularity assessment, providing objective estimates that align with expert pathologists and allowing exploration of biologically relevant patterns such as age-related cellularity changes (66). Tools such as MarrowQuant automate the identification of hematopoietic cells, adipocytes, bone, and vascular structures, reducing user-dependent variability and enabling reproducible mapping of marrow composition across experimental and clinical contexts (67). Similarly, semantic segmentation models trained on expert-annotated slides accurately differentiate hematopoietic from adipose tissue on whole-slide images, offering a standardized and objective method for evaluating cellularity in Philadelphia chromosome-negative MPNs (68).

## AI in bone marrow cytology

AI enables automated, end-to-end analysis of bone marrow cytology by detecting optimal regions on aspirate slides, identifying individual cells, and classifying marrow cell types with high accuracy. The resulting quantitative differential (e.g., a ‘Histogram of Cell Types’) provides a reproducible cytological profile that minimizes inter-observer variability and supports more consistent hematological diagnosis (69). AI-driven digital pathology also facilitates deep learning–based assessment of digitized marrow slides, enabling reliable detection of malignant and reactive abnormalities and promoting more standardized, scalable evaluation workflows (19).

**Figure 2:**
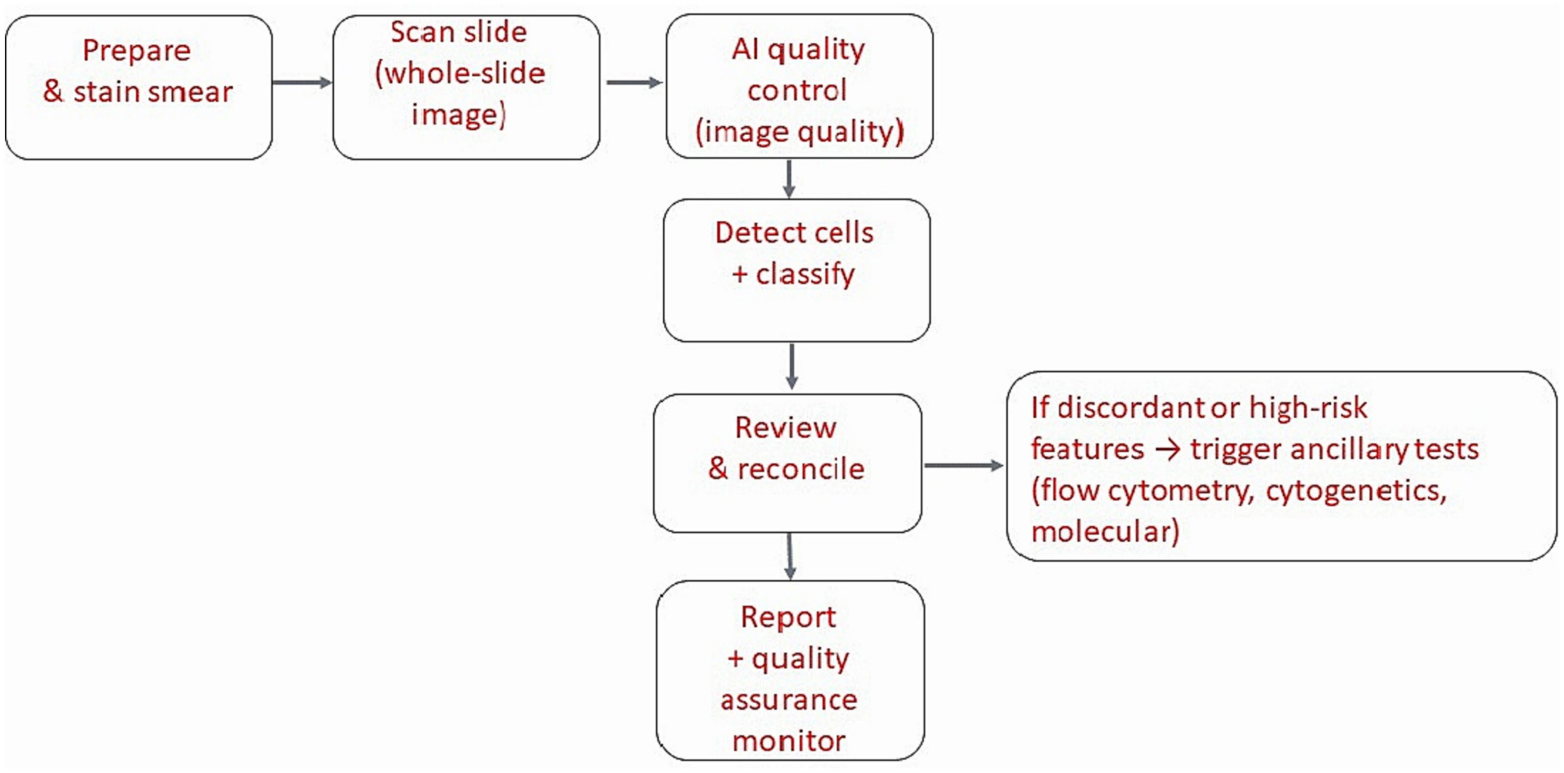
AI-assisted bone marrow aspirate smear cell-type classification integrated into routine clinical workflow.

In routine practice, an implementable bone marrow aspirate (BMA) smear workflow begins with slide digitization (scanner settings and color calibration), followed by automated quality control to exclude thick areas, crush artifact, staining outliers, and poor focus regions The model then performs region selection, cell detection/segmentation, and cell-type classification to generate an automated differential alongside interpretable overlays and uncertainty flags for manual review (70, 71). Outputs can be triaged into concordant cases where AI-assisted differentials support faster sign-out, borderline/discordant cases routed for second review, and suspicious patterns (e.g., excess blasts, atypical promyelocytes, dysplastic forms) that trigger confirmatory testing (flow cytometry, cytogenetics/molecular assays) and structured reporting (72). Figure 2 summarizes these steps and highlights where human verification and ancillary testing remain essential.

Beyond tissue sections, AI-enabled cytomorphology on BMA smears provides a practical example of how computational modalities can be embedded into routine hematology workflows. Using large expert-annotated image datasets, deep neural networks have achieved highly accurate differentiation of bone marrow cell morphologies, supporting automated single-cell classification as a proof-of-concept for routine deployment (73). More recently, externally validated ensemble models have demonstrated expert-level or superior performance across independent centers and scanning systems, supporting generalisable AI-based bone marrow morphometry for hematological diagnosis and quantitative reporting (74).

Deep learning models have also been applied to automate detailed analysis of bone marrow smears in acute myeloid leukemia (AML), including cell segmentation, distinguishing AML from healthy samples, and predicting mutation status (e.g., NPM1) from morphology in research cohorts, which may enhance diagnostic precision (75). AI supports automated identification of hematopoietic lineages and detection of dysplastic cells in MDS, classifying normal and dysplastic forms with high accuracy and reducing the subjectivity of traditional smear evaluation (76). Models such as DysplasiaNet detect hypogranulated dysplastic neutrophils, a challenging but clinically relevant MDS feature, providing more objective granularity assessment and improving consistency in routine laboratory workflows (77). In acute promyelocytic leukemia (APL), AI enables rapid recognition of morphologic patterns associated with the t(15;17) translocation, reliably distinguishing APL from other AML subtypes and from normal marrow, which may be particularly valuable where molecular confirmation is delayed or unavailable (78).

## Limitations of AI in bone marrow biopsy

AI offers substantial advantages in bone marrow biopsy evaluation, yet several limitations constrain its routine clinical adoption. AI models rely heavily on large, well-annotated training datasets, which are difficult to obtain in hematopathology due to disease rarity, inter-institutional variability, and the time-intensive nature of expert annotation (79). Differences in staining quality, slide preparation, and scanning resolution can impair model generalizability, leading to reduced performance across laboratories (80). Many algorithms function as “black boxes,” providing limited interpretability and posing challenges for clinical validation and regulatory approval (81, 82). Additionally, AI systems may overlook rare morphologic patterns or artifacts not well represented in training data (3, 19). Integration into clinical workflows requires robust computational infrastructure, quality control, and ongoing monitoring to prevent drift and ensure reproducibility (83, 84). These limitations highlight the need for standardized datasets, transparent algorithms, and multidisciplinary oversight to ensure safe and reliable deployment of AI in bone marrow biopsy diagnostics.

## Limitations of omics in bone marrow biopsy

Omics technologies have transformed bone marrow diagnostics, yet several limitations hinder their widespread clinical implementation. High-throughput genomic, epigenomic, transcriptomic, proteomic, and metabolomic assays are often costly, technically complex, and require specialized laboratory infrastructure that may not be accessible in all healthcare settings (85, 86). Variability in sample quality, particularly in small or hypocellular marrow biopsies, can affect data integrity and lead to incomplete or biased molecular profiles (87). Many omics datasets are high dimensional, requiring sophisticated bioinformatics expertise and computational resources to interpret, and results may vary depending on analytical pipelines and reference databases (88–90). Moreover, the integration of multi-omics data remains challenging due to differences in temporal resolution, dynamic biological processes, and limited consensus on standardized interpretation frameworks (13, 91). Finally, the clinical significance of certain molecular alterations may be unclear, leading to potential overinterpretation or uncertainty in diagnostic and prognostic decision-making (14, 92). These limitations underscore the need for harmonized protocols, improved analytic tools, and evidence-based validation to ensure reliable and clinically meaningful use of omics in bone marrow biopsy evaluation.

## Future directions

The future of bone marrow biopsy diagnostics lies in the integrated application of omics technologies and AI, enabling a shift from descriptive morphology toward data-driven, precision hematopathology (24, 93, 94). Multi-omics profiling will increasingly be applied directly to bone marrow specimens to capture the complex molecular architecture of hematologic diseases and their microenvironment (95). These approaches will support refined disease classification, early detection of clonal evolution, minimal residual disease monitoring, and improved prediction of therapeutic response (96).

AI will play a central role in integrating and interpreting high-dimensional omics data alongside digital histopathology (97, 98). Deep learning models will increasingly link morphologic patterns in whole-slide images with underlying molecular signatures, enabling virtual molecular profiling from routine H&E or immunohistochemistry (99–101). Spatially resolved omics combined with AI-driven image analysis will allow precise mapping of genetic and proteomic alterations within specific marrow niches, improving understanding of tumor–stroma interactions, immune dysregulation, and treatment resistance (102, 103). Importantly, recent single-cell–resolved spatial mapping of human bone marrow using archival specimens has demonstrated that clinically relevant hematopoietic organization and niche biology can be recovered from pathology archives, including aging-associated remodeling of marrow topography and microvascular–cellular relationships (104).

Future workflows are expected to adopt end-to-end computational pathology pipelines, in which AI automates slide quality control, region selection, cell segmentation, and feature extraction, while multi-omics data provide complementary biological context (105–108). Federated learning and multi-center model training will enhance generalizability, address data-sharing constraints, and support regulatory approval (109). Additionally, explainable AI approaches will improve transparency, enabling pathologists to understand how morphologic and molecular features drive algorithmic predictions (110, 111).

Clinically, these advances will support personalized risk stratification and treatment planning, particularly in complex entities such as myelodysplastic syndromes, myeloproliferative neoplasms, and plasma cell dyscrasias (112). As costs decline and standardization improves, AI-integrated multi-omics bone marrow analysis is expected to transition from a research tool to a routine component of diagnostic and prognostic assessment, positioning the bone marrow biopsy as a central hub for precision hematology (113). These spatial multi-omic atlases of human bone marrow strengthen the case for cross-platform strategies in which spatial proteomics (e.g., CODEX) is co-registered with transcriptomic readouts to deliver clinically interpretable, niche-level biomarkers and standardized, reproducible neighborhood metrics (114).

## Conclusion

Bone marrow biopsy is undergoing a fundamental transformation driven by the convergence of omics technologies and AI. Together, these approaches extend diagnostic evaluation beyond morphology, enabling multidimensional characterization of genetic alterations, cellular heterogeneity, microenvironmental interactions, and disease dynamics. While important challenges remain, including technical complexity, interpretative standardization, and clinical validation, the integration of molecular profiling with AI-driven analytics represents a pivotal shift toward precision hematology. As these technologies mature and become increasingly standardized and accessible, they are poised to enhance diagnostic accuracy, refine prognostication, and support individualized therapeutic strategies, redefining the role of bone marrow biopsy as a central platform for integrated hematologic diagnosis and disease monitoring.

## References

[1] Burkhardt R Frisch B Bartl R. Bone biopsy in haematological disorders. J Clin Pathol. (1982) 35:257–84. doi: 10.1136/jcp.35.3.257, 7040489 PMC497529

[2] Weinberg OK Hasserjian RP. The current approach to the diagnosis of myelodysplastic syndromes. Semin Hematol. (2019) 56:15–21. doi: 10.1053/j.seminhematol.2018.05.015, 30573039

[3] Lewis JE Pozdnyakova O. Digital assessment of peripheral blood and bone marrow aspirate smears. Int J Lab Hematol. (2023) 45:50–8. doi: 10.1111/ijlh.14082, 37211430

[4] Ng GTE Phang SC Yu KS Tiwari L Khurram SA Sloan P . Understanding interobserver variability of pathologists to improve oral epithelial dysplasia grading. Oral Dis. (2025) 31:838–45. doi: 10.1111/odi.15078, 39039698 PMC12021307

[5] Burns J Wilding CP Jones RL Huang PH. Proteomic research in sarcomas: current status and future opportunities. Semin Cancer Biol. (2020) 61:56–70. doi: 10.1016/j.semcancer.2019.11.003, 31722230 PMC7083238

[6] Ray-Coquard I Montesco MC Coindre JM Dei Tos AP Lurkin A Ranchère-Vince D . Sarcoma: concordance between initial diagnosis and centralized expert review in a population-based study within three European regions. Ann Oncol. (2012) 23:2442–9. doi: 10.1093/annonc/mdr610 22331640 PMC3425368

[7] Kilpatrick SE. Keeping it real: merging traditional and contemporary practices in musculoskeletal pathology. Hum Pathol. (2024) 147:1–4. doi: 10.1016/j.humpath.2024.03.007 38556003

[8] Suster D Suster S. Genetic characteristics and molecular diagnostics of bone tumors. J Cancer Metastasis Treat. (2021) 7:8. doi: 10.20517/2394-4722.2020.119

[9] Walsh E Orsi NM. The current troubled state of the global pathology workforce: a concise review. Diagn Pathol. (2024) 19:163. doi: 10.1186/s13000-024-01590-2, 39709433 PMC11662708

[10] Barberis M. Shortage of pathologists: a candid narrative. Pathologica. (2025) 117:452–4. doi: 10.32074/1591-951X-N1613, 41243518 PMC12620944

[11] Cai W Jiang L Zhao C Zhou X. Advances in omics technologies for traditional Chinese medicine in the prevention and treatment of metabolic bone diseases. Front Pharmacol. (2025) 16:1576286. doi: 10.3389/fphar.2025.1576286, 40290428 PMC12021879

[12] Heo YJ Hwa C Lee GH Park JM An JY. Integrative multi-omics approaches in cancer research: from biological networks to clinical subtypes. Mol Cells. (2021) 44:433–43. doi: 10.14348/molcells.2021.0042, 34238766 PMC8334347

[13] Chakraborty S Sharma G Karmakar S Banerjee S. Multi-omics approaches in cancer biology: new era in cancer therapy. Biochim Biophys Acta Mol basis Dis. (2024) 1870:167120. doi: 10.1016/j.bbadis.2024.167120, 38484941

[14] Chen C Wang J Pan D Wang X Xu Y Yan J . Applications of multi-omics analysis in human diseases. MedComm. (2023) 4:e315. doi: 10.1002/mco2.315, 37533767 PMC10390758

[15] Yi S Yan Y Jin M Bhattacharya S Wang Y Wu Y . Genomic and transcriptomic profiling reveals distinct molecular subsets associated with outcomes in mantle cell lymphoma. J Clin Invest. (2022) 132:e153283. doi: 10.1172/JCI153283, 34882582 PMC8803323

[16] Hu Y Luo Y Tang G Huang Y Kang J Wang D. Artificial intelligence and its applications in digital hematopathology. Blood Sci. (2022) 4:136–42. doi: 10.1097/BS9.0000000000000130, 36518598 PMC9742095

[17] Haferlach T Pohlkamp C Heo I Drescher R Hänselmann S Lörch T . Automated peripheral blood cell differentiation using artificial intelligence: a study with more than 10,000 routine samples in a specialized leukemia laboratory. Blood. (2021) 138:103. doi: 10.1182/blood-2021-152447

[18] Wang CW Huang SC Lee YC Shen YJ Meng SI Gaol JL. Deep learning for bone marrow cell detection and classification on whole-slide images. Med Image Anal. (2022) 75:102270. doi: 10.1016/j.media.2021.102270, 34710655

[19] El Achi H Khoury JD. Artificial intelligence and digital microscopy applications in diagnostic hematopathology. Cancers (Basel). (2020) 12:797. doi: 10.3390/cancers12040797 32224980 PMC7226574

[20] Baranova K Tran C Plantinga P Sangle N. Evaluation of an open-source machine-learning tool to quantify bone marrow plasma cells. J Clin Pathol. (2021) 74:462–8. doi: 10.1136/jclinpath-2021-207524, 33952591

[21] van Eekelen L Litjens G Hebeda KM. Artificial intelligence in bone marrow histological diagnostics: potential applications and challenges. Pathobiology. (2024) 91:8–17. doi: 10.1159/000529701, 36791682 PMC10937040

[22] Ryou H Lomas O Theissen H Thomas E Rittscher J Royston D. Quantitative interpretation of bone marrow biopsies in MPN—what’s the point in a molecular age? Br J Haematol. (2023) 203:523–35. doi: 10.1111/bjh.19154, 37858962 PMC10952168

[23] Walter W Haferlach C Nadarajah N Schmidts I Kühn C Kern W . How artificial intelligence might disrupt diagnostics in hematology in the near future. Oncogene. (2021) 40:4271–80. doi: 10.1038/s41388-021-01861-y, 34103684 PMC8225509

[24] Isaac A Klontzas ME Dalili D Akdogan AI Fawzi M Gugliemi G . Revolutionising osseous biopsy: the impact of artificial intelligence in the era of personalized medicine. Br J Radiol. (2025) 98:795–809. doi: 10.1093/bjr/tqaf018, 39878877 PMC12089761

[25] Obeagu EI. Revolutionizing hematological disorder diagnosis: unraveling the role of artificial intelligence. Ann Med Surg (Lond). (2025) 87:3445–57. doi: 10.1097/MS9.0000000000003227, 40486570 PMC12140674

[26] Calciolari E Donos N. The use of omics profiling to improve outcomes of bone regeneration and osseointegration: how far are we from personalized medicine in dentistry? J Proteome. (2018) 188:85–96. doi: 10.1016/j.jprot.2018.01.017, 29410240

[27] Yang L Xu Z Liu J Chang X Ren Z Xiao W. Multi-omics insights into bone tissue injury and healing: bridging orthopedic trauma and regenerative medicine. Burns Trauma. (2025) 13:tkaf019. doi: 10.1093/burnst/tkaf019 40438296 PMC12118463

[28] Kersey AL Nguyen T-U Nayak B Singh I Gaharwar AK. Omics-based approaches to guide the design of biomaterials. Mater Today. (2023) 64:98–120. doi: 10.1016/j.mattod.2023.01.018

[29] Hickman TT Rathan-Kumar S Peck SH. Development, pathogenesis, and regeneration of the intervertebral disc: current and future insights spanning traditional to omics methods. Front Cell Dev Biol. (2022) 10:841831. doi: 10.3389/fcell.2022.841831, 35359439 PMC8963184

[30] Yang J Wu J. Discovery of potential biomarkers for osteoporosis diagnosis by individual omics and multi-omics technologies. Expert Rev Mol Diagn. (2023) 23:505–20. doi: 10.1080/14737159.2023.2208750, 37140363

[31] Tewary M Shakiba N Zandstra PW. Stem cell bioengineering: building from stem cell biology. Nat Rev Genet. (2018) 19:595–614. doi: 10.1038/s41576-018-0040-z 30089805

[32] Abazari R Mahjoub AR Shariati J. Synthesis of a nanostructured pillar MOF with high adsorption capacity towards antibiotics pollutants from aqueous solution. J Hazard Mater. (2019) 366:439–51. doi: 10.1016/j.jhazmat.2018.12.030, 30562656

[33] Ziegenhain C Vieth B Parekh S Hellmann I Enard W. Quantitative single-cell transcriptomics. Brief Funct Genomics. (2018) 17:220–32. doi: 10.1093/bfgp/ely009, 29579145 PMC6063296

[34] Kinaret PAS Serra A Federico A Kohonen P Nymark P Liampa I . Transcriptomics in toxicogenomics, part I: experimental design, technologies, publicly available data, and regulatory aspects. Nano. (2020) 10:750. doi: 10.3390/nano10040750, 32326418 PMC7221878

[35] Piunti A Shilatifard A. Epigenetic balance of gene expression by Polycomb and COMPASS families. Science. (2016) 352:aad9780. doi: 10.1126/science.aad9780, 27257261

[36] Ren S Li J Dorado J Sierra A González-Díaz H Duardo A . From molecular mechanisms of prostate cancer to translational applications: based on multi-omics fusion analysis and intelligent medicine. Health Inf Sci Syst. (2023) 12:6. doi: 10.1007/s13755-023-00264-5, 38125666 PMC10728428

[37] Neri S. Genetic stability of mesenchymal stromal cells for regenerative medicine applications: a fundamental biosafety aspect. Int J Mol Sci. (2019) 20:2406. doi: 10.3390/ijms20102406, 31096604 PMC6566307

[38] Santos-Moreno J Schaerli Y. CRISPR-based gene expression control for synthetic gene circuits. Biochem Soc Trans. (2020) 48:1979–93. doi: 10.1042/BST20200020, 32964920 PMC7609024

[39] Sethi S Brietzke E. Recent advances in lipidomics: analytical and clinical perspectives. Prostaglandins Other Lipid Mediat. (2017) 128:8–16. doi: 10.1016/j.prostaglandins.2016.12.002, 28039059

[40] Wu Z Bagarolo GI Thoröe-Boveleth S Jankowski J. Lipidomics: mass spectrometric and chemometric analyses of lipids. Adv Drug Deliv Rev. (2020) 159:294–307. doi: 10.1016/j.addr.2020.06.009, 32553782

[41] Vvedenskaya O Holčapek M Vogeser M Ekroos K Meikle PJ Bendt AK. Clinical lipidomics: a community-driven roadmap to translate research into clinical applications. J Mass Spectrom Adv Clin Lab. (2022) 24:1–4. doi: 10.1016/j.jmsacl.2022.02.002, 35199094 PMC8844780

[42] Xu J Li Z Tower RJ Negri S Wang Y Meyers CA . NGF–p75 signaling coordinates skeletal cell migration during bone repair. Sci Adv. (2022) 8:eabl5716. doi: 10.1126/sciadv.abl5716, 35302859 PMC8932666

[43] Sivaraj KK Majev P-G Jeong H-W Dharmalingam B Zeuschner D Schröder S . Mesenchymal stromal cell–derived septoclasts resorb cartilage during developmental ossification and fracture healing. Nat Commun. (2022) 13:571. doi: 10.1038/s41467-022-28142-w, 35091558 PMC8799643

[44] Wang Y Wang Q Xu Q Li J Zhao F. Single-cell RNA sequencing analysis dissected the osteo-immunology microenvironment and revealed key regulators in osteoporosis. Int Immunopharmacol. (2022) 113:109302. doi: 10.1016/j.intimp.2022.109302, 36257255

[45] Tower RJ Li Z Cheng Y-H Wang X-W Rajbhandari L Zhang Q . Spatial transcriptomics reveals a role for sensory nerves in preserving cranial suture patency through modulation of BMP/TGF-β signaling. Proc Natl Acad Sci USA. (2021) 118:e2103087118. doi: 10.1073/pnas.2103087118, 34663698 PMC8545472

[46] Yang Y Yang M Shi D Chen K Zhao J He S . Single-cell RNA sequencing reveals cellular landscape–specific characteristics and potential etiologies for adolescent idiopathic scoliosis. JOR Spine. (2021) 4:e1184. doi: 10.1002/jsp2.1184 35005449 PMC8717101

[47] Lamas A Regal P Vázquez B Miranda JM Franco CM Cepeda A. Transcriptomics: a powerful tool to evaluate the behavior of foodborne pathogens in the food production chain. Food Res Int. (2019) 125:108543. doi: 10.1016/j.foodres.2019.108543, 31554082

[48] Rike WA Stern S. Proteins and transcriptional dysregulation of the brain extracellular matrix in Parkinson’s disease: a systematic review. Int J Mol Sci. (2023) 24:7435. doi: 10.3390/ijms24087435, 37108598 PMC10138539

[49] Song Y Soto J Chen B Yang L Li S. Cell engineering: biophysical regulation of the nucleus. Biomaterials. (2020) 234:119743. doi: 10.1016/j.biomaterials.2019.119743 31962231

[50] Bastounis EE Yeh Y-T Theriot JA. Subendothelial stiffness alters endothelial cell traction force generation while exerting a minimal effect on the transcriptome. Sci Rep. (2019) 9:18209. doi: 10.1038/s41598-019-54336-2, 31796790 PMC6890669

[51] Gaharwar AK Singh I Khademhosseini A. Engineered biomaterials for in situ tissue regeneration. Nat Rev Mater. (2020) 5:686–705. doi: 10.1038/s41578-020-0209-x

[52] Tsiridis E Giannoudis PV. Transcriptomics and proteomics: advancing the understanding of the genetic basis of fracture healing. Injury. (2006) 37:S13–9. doi: 10.1016/j.injury.2006.02.036 16616752

[53] Gonzalez-Covarrubias V Martínez-Martínez E Del Bosque-Plata L. The potential of metabolomics in biomedical applications. Meta. (2022) 12:194. doi: 10.3390/metabo12020194, 35208267 PMC8880031

[54] Kogut MH Lee A Santin E. Microbiome and pathogen interaction with the immune system. Poult Sci. (2020) 99:1906–13. doi: 10.1016/j.psj.2019.12.011, 32241470 PMC7587753

[55] Levy M Blacher E Elinav E. Microbiome, metabolites and host immunity. Curr Opin Microbiol. (2017) 35:8–15. doi: 10.1016/j.mib.2016.10.003, 27883933

[56] Gowda GN Zhang S Gu H Asiago V Shanaiah N Raftery D. Metabolomics-based methods for early disease diagnostics. Expert Rev Mol Diagn. (2008) 8:617–33. doi: 10.1586/14737159.8.5.617, 18785810 PMC3890417

[57] Wang R Li B Lam SM Shui G. Integration of lipidomics and metabolomics for in-depth understanding of cellular mechanisms and disease progression. J Genet Genomics. (2020) 47:69–83. doi: 10.1016/j.jgg.2019.11.009, 32178981

[58] Johnson CH Ivanisevic J Siuzdak G. Metabolomics: beyond biomarkers and towards mechanisms. Nat Rev Mol Cell Biol. (2016) 17:451–9. doi: 10.1038/nrm.2016.25, 26979502 PMC5729912

[59] Yu D Zhang H Song Y Tao Y Zhou F Wang Z . Artificial intelligence–based quantitative bone marrow pathology analysis for myeloproliferative neoplasms. Haematologica. (2025) 110:2691–701. doi: 10.3324/haematol.2024.286123, 40501395 PMC12580698

[60] Gedefaw L Liu CF Ip RKL Tse HF Yeung MHY Yip SP . Artificial intelligence–assisted diagnostic cytology and genomic testing for hematologic disorders. Cells. (2023) 12:1755. doi: 10.3390/cells12131755, 37443789 PMC10340428

[61] Ryou H Sirinukunwattana K Aberdeen A Grindstaff G Stolz BJ Byrne H . Continuous indexing of fibrosis (CIF): improving the assessment and classification of MPN patients. Leukemia. (2023) 37:348–58. doi: 10.1038/s41375-022-01773-0, 36470992 PMC9898027

[62] Sirinukunwattana K Aberdeen A Theissen H Sousos N Psaila B Mead AJ . Artificial intelligence–based morphological fingerprinting of megakaryocytes: a new tool for assessing disease in MPN patients. Blood Adv. (2020) 4:3284–94. doi: 10.1182/bloodadvances.2020002230, 32706893 PMC7391156

[63] Brück OE Lallukka-Brück SE Hohtari HR Ianevski A Ebeling FT Kovanen PE . Machine learning of bone marrow histopathology identifies genetic and clinical determinants in patients with MDS. Blood Cancer Discov. (2021) 2:238–49. doi: 10.1158/2643-3230.BCD-20-0162, 34661156 PMC8513905

[64] Fu F Guenther A Sakhdari A McKee TD Xia D. Deep learning accurately quantifies plasma cell percentages on CD138-stained bone marrow samples. J Pathol Inform. (2022) 13:100011. doi: 10.1016/j.jpi.2022.100011, 35242448 PMC8873946

[65] Song TH Sanchez V ElDaly H Rajpoot NM. Simultaneous cell detection and classification in bone marrow histology images. IEEE J Biomed Health Inform. (2019) 23:1469–76. doi: 10.1109/JBHI.2018.2878945 30387756

[66] van Eekelen L Pinckaers H van den Brand M Hebeda KM Litjens G. Using deep learning for quantification of cellularity and cell lineages in bone marrow biopsies and comparison to normal age-related variation. Pathology. (2022) 54:318–27. doi: 10.1016/j.pathol.2021.07.011, 34772487

[67] Tratwal J Bekri D Boussema C Sarkis R Kunz N Koliqi T . MarrowQuant across aging and aplasia: a digital pathology workflow for quantification of bone marrow compartments in histological sections. Front Endocrinol (Lausanne). (2020) 11:480. doi: 10.3389/fendo.2020.00480, 33071956 PMC7542184

[68] D’Abbronzo G D’Antonio A De Chiara A Panico L Sparano L Diluvio A . Development of an artificial intelligence–based tool for automated assessment of cellularity in bone marrow biopsies in Ph-negative myeloproliferative neoplasms. Cancers (Basel). (2024) 16:1687. doi: 10.3390/cancers16091687, 38730640 PMC11083301

[69] Tayebi RM Mu Y Dehkharghanian T Ross C Sur M Foley R . Automated bone marrow cytology using deep learning to generate a histogram of cell types. Commun Med. (2022) 2:45. doi: 10.1038/s43856-022-00107-6, 35603269 PMC9053230

[70] Chandradevan R Aljudi AA Drumheller BR Kunananthaseelan N Amgad M Gutman DA . Machine-based detection and classification for bone marrow aspirate differential counts: initial development focusing on nonneoplastic cells. Lab Investig. (2020) 100:98–109. doi: 10.1038/s41374-019-0325-7, 31570774 PMC6920560

[71] Lewis JE Shebelut CW Drumheller BR Zhang X Shanmugam N Attieh M . An automated pipeline for differential cell counts on whole-slide bone marrow aspirate smears. Mod Pathol. (2023) 36:100003. doi: 10.1016/j.modpat.2022.100003, 36853796 PMC10310355

[72] Zhou S Ran L Yao Y Wu X Liu Y Wang C . VFM-SSL-BMADCC-framework: vision foundation model and self-supervised learning based automated framework for differential cell counts on whole-slide bone marrow aspirate smears. Front Med (Lausanne). (2025) 12:1624683. doi: 10.3389/fmed.2025.1624683, 41070066 PMC12504877

[73] Matek C Krappe S Münzenmayer C Haferlach T Marr C. Highly accurate differentiation of bone marrow cell morphologies using deep neural networks on a large image data set. Blood. (2021) 138:1917–27. doi: 10.1182/blood.2020010568, 34792573 PMC8602932

[74] Sun S Yin Z Van Cleave JG Wang L Fried B Bilal KH . DeepHeme, a high-performance, generalizable deep ensemble for bone marrow morphometry and hematologic diagnosis. Sci Transl Med. (2025) 17:eadq2162. doi: 10.1126/scitranslmed.adq2162 40498857

[75] Eckardt JN Middeke JM Riechert S Schmittmann T Sulaiman AS Kramer M . Deep learning detects acute myeloid leukemia and predicts NPM1 mutation status from bone marrow smears. Leukemia. (2022) 36:111–8. doi: 10.1038/s41375-021-01408-w, 34497326 PMC8727290

[76] Lee N Jeong S Park MJ Song W. Deep learning application of the discrimination of bone marrow aspiration cells in patients with myelodysplastic syndromes. Sci Rep. (2022) 12:18677. doi: 10.1038/s41598-022-21887-w, 36333407 PMC9636228

[77] Acevedo A Merino A Boldú L Molina Á Alférez S Rodellar J. A convolutional neural network predictive model for automatic recognition of hypogranulated neutrophils in myelodysplastic syndromes. Comput Biol Med. (2021) 134:104479. doi: 10.1016/j.compbiomed.2021.104479 34010795

[78] Eckardt JN Schmittmann T Riechert S Kramer M Sulaiman AS Sockel K . Deep learning identifies acute promyelocytic leukemia in bone marrow smears. BMC Cancer. (2022) 22:201. doi: 10.1186/s12885-022-09307-8, 35193533 PMC8864866

[79] Liao H Zhang F Chen F Li Y Sun Y Sloboda DD . Application of artificial intelligence in laboratory hematology: advances, challenges, and prospects. Acta Pharm Sin B. (2025) 15:5702–33. doi: 10.1016/j.apsb.2025.05.036, 41311404 PMC12648047

[80] Komura D Ochi M Ishikawa S. Machine learning methods for histopathological image analysis: updates in 2024. Comput Struct Biotechnol J. (2024) 27:383–400. doi: 10.1016/j.csbj.2024.12.033, 39897057 PMC11786909

[81] Olawade DB Adeniji YJ Olatunbosun FA Egbon E David-Olawade AC. Artificial intelligence for anemia screening, diagnosis, and management: a narrative review. Curr Res Transl Med. (2025):103560. doi: 10.1016/j.retram.2025.103560 41401691

[82] Syrykh C van den Brand M Kather JN Laurent C. Role of artificial intelligence in haematolymphoid diagnostics. Histopathology. (2025) 86:58–68. doi: 10.1111/his.15327, 39435690 PMC11648359

[83] Yoon S Hur M Lee GH Nam M Kim H. How reproducible is the data from Sysmex DI-60 in leukopenic samples? Diagnostics (Basel). (2021) 11:2173. doi: 10.3390/diagnostics11122173, 34943409 PMC8700691

[84] Lapić I Miloš M Dorotić M Drenški V Coen Herak D Rogić D. Analytical validation of white blood cell differential and platelet assessment on the Sysmex DI-60 digital morphology analyzer. Int J Lab Hematol. (2023) 45:668–77. doi: 10.1111/ijlh.14101, 37255419

[85] Karahalil B. Overview of systems biology and omics technologies. Curr Med Chem. (2016) 23:4221–30. doi: 10.2174/0929867323666160926150617, 27686657

[86] Magro D Venezia M Balistreri CR. The omics technologies and liquid biopsies: advantages, limitations, applications. Med. Omics. (2024) 11:100039. doi: 10.1016/j.meomic.2024.100039

[87] Janiesch C Zschech P Heinrich K. Machine learning and deep learning. Electron Mark (Dusseldorf). (2021) 31:685–95. doi: 10.1007/s12525-021-00475-2

[88] Subramanian I Verma S Kumar S Jere A Anamika K. Multi-omics data integration, interpretation, and its application. Bioinform Biol Insights. (2020) 14:1177932219899051. doi: 10.1177/1177932219899051, 32076369 PMC7003173

[89] Vitorino R. Transforming clinical research: the power of high-throughput omics integration. Proteomes. (2024) 12:25. doi: 10.3390/proteomes12030025, 39311198 PMC11417901

[90] Yetgin A. Revolutionizing multi-omics analysis with artificial intelligence and data processing. Quant Biol. (2025) 13:e70002. doi: 10.1002/qub2.70002, 41675959 PMC12806145

[91] Mohr AE Ortega-Santos CP Whisner CM Klein-Seetharaman J Jasbi P. Navigating challenges and opportunities in multi-omics integration for personalized healthcare. Biomedicine. (2024) 12:1496. doi: 10.3390/biomedicines12071496, 39062068 PMC11274472

[92] Liang A Kong Y Chen Z Qiu Y Wu Y Zhu X . Advancements and applications of single-cell multi-omics techniques in cancer research: unveiling heterogeneity and paving the way for precision therapeutics. Biochem Biophys Rep. (2023) 37:101589. doi: 10.1016/j.bbrep.2023.101589, 38074997 PMC10698529

[93] Nazha A Elemento O Ahuja S Lam B Miles M Shouval R . Artificial intelligence in hematology. Blood. (2025) 146:2283–92. doi: 10.1182/blood.2025029876, 40845137

[94] Mandefro B Berta DM Kelem A Teketelew BB Melkamu A Much Y . The role of advanced diagnostics on precision medicine in hemato oncology. Discov Oncol. (2025) 16:1525. doi: 10.1007/s12672-025-03169-9, 40788517 PMC12339839

[95] Alhamrani SQ Ball GR El-Sherif AA Ahmed S Mousa NO Alghorayed SA . Machine learning for multi-omics characterization of blood cancers: a systematic review. Cells. (2025) 14:1385. doi: 10.3390/cells14171385, 40940796 PMC12427946

[96] Soleimani Samarkhazan H. Integrating multi-omics approaches in acute myeloid leukemia (AML): advancements and clinical implications. Clin Exp Med. (2025) 25:311. doi: 10.1007/s10238-025-01858-x, 40886194 PMC12399717

[97] Phan NN Chattopadhyay A Chuang EY. Role of artificial intelligence in integrated analysis of multi-omics and imaging data in cancer research. Transl Cancer Res. (2019) 8:E7–E10. doi: 10.21037/tcr.2019.12.17, 35117055 PMC8797959

[98] Marouf AA Rokne JG Alhajj R. Integrating multi-omics and medical imaging in artificial intelligence–based cancer research: an umbrella review of fusion strategies and applications. Cancers (Basel). (2025) 17:3638. doi: 10.3390/cancers17223638, 41301005 PMC12650882

[99] Coudray N Tsirigos A. Deep learning links histology, molecular signatures and prognosis in cancer. Nat Cancer. (2020) 1:755–7. doi: 10.1038/s43018-020-0099-2, 35122048 PMC11330634

[100] Frascarelli C Venetis K Marra A Mane E Ivanova M Cursano G . Deep learning algorithm on H&E whole-slide images to characterize TP53 alterations frequency and spatial distribution in breast cancer. Comput Struct Biotechnol J. (2024) 23:4252–9. doi: 10.1016/j.csbj.2024.11.037, 39678362 PMC11638532

[101] Venturi F Veronesi G Gualandi A Magnaterra E Scotti B Sotiri I . From slide to insight: the emerging alliance of digital pathology and AI in melanoma diagnostics. Cancers (Basel). (2025) 17:3696. doi: 10.3390/cancers17223696, 41301061 PMC12651191

[102] Manogaran S Ramadoss R Selvam SP Sundar S Krishnasamy N Hemashree . Artificial intelligence–driven spatial transcriptomics in oral squamous cell carcinoma: mapping the tumor microenvironment and personalizing therapy. J Oral Biol Craniofac Res. (2025) 15:1862–73. doi: 10.1016/j.jobcr.2025.10.015 41282271 PMC12639440

[103] Cheng X Peng T Chu T Yang Y Liu J Gao Q . Application of single-cell and spatial omics in deciphering cellular hallmarks of cancer drug response and resistance. J Hematol Oncol. (2025) 18:70. doi: 10.1186/s13045-025-01722-1, 40605007 PMC12224702

[104] Sarachakov A Varlamova A Svekolkin V Polyakova M Valencia I Unkenholz C . Spatial mapping of human hematopoiesis at single-cell resolution reveals aging-associated topographic remodeling. Blood. (2024) 144:464. doi: 10.1182/blood.2024025832 37774374

[105] Pati P Karkampouna S Bonollo F Compérat E Radić M Spahn M . Accelerating histopathology workflows with generative AI–based virtually multiplexed tumour profiling. Nat Mach Intell. (2024) 6:1077–93. doi: 10.1038/s42256-024-00889-5, 39309216 PMC11415301

[106] Sweileh MW. AI-powered histopathology slide image interpretation in oncology: a comprehensive knowledge mapping and bibliometric analysis. Digit Health. (2025) 11:20552076251393286. doi: 10.1177/20552076251393286, 41181553 PMC12576219

[107] Huo T Wu W Chen X Xue M Liu P Zhang J . Deep learning–based multimodal data fusion in bone tumor management: advances in clinical decision support. Intell Oncol. (2025) 1:204–15. doi: 10.1016/j.intonc.2025.06.005

[108] Mehmood S Zubair M Khan FM Shah AA Abbas S Adnan KM. Deep learning in bone marrow cytomorphology: advances in segmentation, classification, and clinical translation. Med Oncol. (2026) 43:22. doi: 10.1007/s12032-025-03127-z, 41284066

[109] Ghete T Kock F Pontones M Pfrang D Westphal M Höfener H . Models for the marrow: a comprehensive review of AI-based cell classification methods and malignancy detection in bone marrow aspirate smears. Hemasphere. (2024) 8:e70048. doi: 10.1002/hem3.70048, 39629240 PMC11612571

[110] Walter W Pohlkamp C Meggendorfer M Nadarajah N Kern W Haferlach C . Artificial intelligence in hematological diagnostics: game changer or gadget? Blood Rev. (2023) 58:101019. doi: 10.1016/j.blre.2022.101019, 36241586

[111] Rao BD Madhavi K. Enhancing bone cancer detection through optimized pretrained deep learning models and explainable AI using the osteosarcoma tumor assessment dataset. Sci Rep. (2025) 15:39104. doi: 10.1038/s41598-025-26051-8, 41203786 PMC12595057

[112] Molla G Bitew M. Revolutionizing personalized medicine: synergy with multi-omics data generation, main hurdles, and future perspectives. Biomedicine. (2024) 12:2750. doi: 10.3390/biomedicines12122750, 39767657 PMC11673561

[113] Hsu CY Askar S Alshkarchy SS Nayak PP Attabi KAL Khan MA . AI-driven multi-omics integration in precision oncology: bridging the data deluge to clinical decisions. Clin Exp Med. (2025) 26:29. doi: 10.1007/s10238-025-01965-9, 41266662 PMC12634751

[114] Bandyopadhyay S Duffy MP Ahn KJ Sussman JH Pang M Smith D . Mapping the cellular biogeography of human bone marrow niches using single-cell transcriptomics and proteomic imaging. Cell. (2024) 187:3120–3140.e29. doi: 10.1016/j.cell.2024.04.013, 38714197 PMC11162340

